# Development of a questionnaire to assess the impact on parents of their infant’s bronchiolitis hospitalization

**DOI:** 10.1186/1472-6963-13-272

**Published:** 2013-07-12

**Authors:** Alexandre Lapillonne, Antoine Regnault, Véronique Gournay, Jean-Bernard Gouyon, Khadra Benmedjahed, Daniela Anghelescu, Benoit Arnould, Guy Moriette

**Affiliations:** 1 APHP Brune-Necker Hospital, Paris Descartes University, Paris, France; 2 Mapi, Lyon, France; 3 Centre Hospitalier Universitaire, Nantes, France; 4 Department of Paediatrics, Dijon University Hospital, France; GHSR, CIC-EC, CHR de la Réunion, Reunion Island, France; 5 Abbvie France, Rungis, France; 6 Service de Médecine Néonatale de Port-Royal, Groupe Hospitalier Cochin, AP-HP, Paris, France; 7 Faculté de Médecine, Université Paris Descartes, Paris, France

**Keywords:** Bronchiolitis, Questionnaires, Family, Infant care, Hospitalization

## Abstract

**Background:**

Bronchiolitis is a distressing respiratory condition and the most common cause of hospitalization during the first year of life. The hospitalization of an infant is a stressful event for parents and deserves careful consideration. The objective of this work was to develop and validate a self-administered instrument that comprehensively assesses the impact on parents of the hospitalization of their infant for bronchiolitis.

**Methods:**

The Impact of Bronchiolitis Hospitalization Questionnaire (IBHQ^©^) was developed using a literature review and pre-study interviews with both parents and clinicians. For finalization and psychometric validation, it was included in a multicenter, longitudinal, observational study conducted in France. Parents of infants under the age of 1 year and hospitalized for bronchiolitis were asked to complete the questionnaire at hospital discharge, and 3 months after.

**Results:**

Seven hundred and seven questionnaires were completed by the parents of the 463 eligible infants. After finalization, based on principal component analyses, the IBHQ included 30 core items allowing the calculation of 7 dimension core scores (Worries and distress; Fear for future; Guilt; Impact on daily organization; Physical impact; Impact on behavior with hospitalized infant; Financial impact), as well as 16 optional items, allowing the calculation of 5 optional dimension scores (Disturbed breastfeeding; Physical reaction of hospitalized infant; Impact on feeding; Impact on behavior with other infants; Siblings’ reaction). Internal consistency reliability and construct validity of the IBHQ were satisfactory. The highest impact was observed for “Worries and distress”, “Fear for future” and “Impact on daily organization” scores.

**Conclusions:**

The IBHQ is a reliable and valid instrument for assessing the multifaceted impact on parents of the hospitalization of their infant for bronchiolitis.

## Background

Bronchiolitis is a distressing respiratory condition most often caused by respiratory syncytial virus (RSV), which usually affects infants under 2 years of age [1], and is the most common cause of hospitalization during the first year of life. Hospitalization for bronchiolitis peaks between months 3–6 of life, with a rate of around 1-3% of all infants [2].

The impact of bronchiolitis hospitalization is generally evaluated based on the epidemiologic criterion of risk of complications secondary to hospitalization or the economic criterion of costs associated with hospitalization. However, because the hospitalization of an infant is a stressful event for parents [3,4], their perspective is also particularly important to consider in this evaluation.

The impact of an infant’s illness on his or her family has been studied in several conditions (complex chronic conditions [5], critical illness and injury treated in intensive care units [6], asthma [7]) and from various perspectives (psychosocial impact [8,9], impact on familial functioning [10,11], impact on mental health [12]). The hospital admission of one’s own infant is a very personal experience and therefore the evaluation of its impact from an individual perspective necessitates the involvement of a self-administered questionnaire. While some questionnaires explore the impact of an infant’s illness on the family (Paediatric Asthma Caregiver’s Quality of Life Questionnaire [7], PedsQL Family Impact Module [5], Impact-On Family scale [13], Impact of Childhood Illness Scale [14]), they do not address the specific issues related to infant hospitalization, either in general or in the particular case of admission for bronchiolitis.

Our objective was to develop and validate a self-administered questionnaire that comprehensively assesses the impact on parents of the hospitalization of their infant for bronchiolitis. The primary purpose of this instrument is to allow the experience of parents during the hospitalization of their infant for bronchiolitis, as well as during the months following discharge, to be completely, accurately and specifically described.

## Methods

### Development of the questionnaire

The questionnaire was developed in a stepwise approach consisting of four stages. The first stage aimed to identify the concepts to be measured by the questionnaire. It was based on a literature review targeting the concepts measured or discussed in the scientific literature about the social, psychological and economic impact on parents of various child conditions (including respiratory conditions). The first stage also included interviews with clinicians and parents of children recently hospitalized for bronchiolitis: 3 clinician interviews were performed to capture the clinician perspective on the impact of the hospitalization on parents and 5 exploratory interviews with parents. The information collected during this first phase was critically compiled to create a preliminary conceptual model of the impact on parents of bronchiolitis hospitalization, to be used as a basis for the development of the questionnaire. In the second stage, the conceptual model was tested and finalized after 13 additional exploratory parent interviews. Thirdly, a test version of the questionnaire was developed using parents’ own words. The fourth stage consisted of comprehension tests that were conducted with 9 other parents, to confirm that the questionnaire was comprehensive, understandable, relevant and well-accepted by parents. Importantly, particular caution was given to parent selection at all stages of the development process to capture the heterogeneity of the situations: interviewees were both fathers and mothers, with various personal backgrounds (educational levels, working status, rural or urban) and both parents of full- and preterm infants were included (the description of the parents interviewed during the development process is provided as Additional file 1: Table S1).

The questionnaire resulting from this process included 62 items covering 8 hypothesized dimensions: parents’ emotional reactions, hospitalized infant’s reactions, parent’s physical reactions, daily organization, siblings’ reaction, behavior of the parent with their infant, relationships with the parent’s partner, financial consequences. Questionnaire items had a 4-point Likert-type response scale ranging from “not at all” to “extremely”, with an additional “not applicable/I don’t know” response choice for some of them.

These items were grouped into two distinct sets: 36 core items were strictly focused on the impact of hospitalization on parents and could be answered by all the parents, and 26 optional items that either concerned the infant’s reaction as perceived by the parents (and therefore not the direct impact on parents) or could be completed only in certain cases (i.e., when the infant had siblings, when the parent had a partner, when the infant was breastfed).

As it was assumed that the impact on parents could last even after hospital discharge, two versions of the questionnaire were developed: one version was designed to be completed in the week following the hospital discharge (DC), and the second to be completed 3 months after discharge to assess the impact during the follow-up (FU) period. The two versions included similar items but differed slightly in their wording. The most important difference between the two versions was the reference period: the former referred to the hospitalization period while the latter referred to the period since hospital discharge.

### Study design

To finalize and validate the Impact of Bronchiolitis Hospitalization Questionnaire (IBHQ^©^), a multicenter, prospective, observational study was conducted in France from October 2008 to July 2009. Eighty-six attending physicians experienced in the management of infants hospitalized for bronchiolitis (this convenience sample was composed of 49 pediatricians, 21 neonatologists, 3 pediatric cardiologists and 13 pediatric pneumologists) were asked to recruit 7 infants each: 3 preterm infants (born at ≤ 35 weeks of gestational age), 3 full-terms, and 1 infant with congenital heart disease (CHD).

Inclusion criteria were: infants under the age of 1 year, hospitalized for bronchiolitis only and not participating in another clinical study; parents able to read, understand and complete a questionnaire in French. Children already hospitalized before the bronchiolitis episode for a reason other than bronchiolitis, or whose parents had already participated in this study, were excluded. Infants were recruited at the visit preceding hospital discharge; on this occasion, investigators reported data about the current hospitalization, and the infant’s medical history, including health care received during the neonatal period and risk factors for bronchiolitis. Parents completed the discharge questionnaire within the week after discharge and the follow-up questionnaire three months after discharge. It was asked that the same parent respond to the questionnaires at both discharge and follow-up.

### Statistical analyses

#### General considerations

In order to define the structure of the final questionnaire, data from the discharge and follow-up questionnaire were pooled. This sample was then randomly divided into two independent data sets: a first set (construction sample) used to finalize the questionnaire (i.e. to select items to be retained and define the dimension scores), and a second set (validation sample) used to validate the structure independently. All analyses aiming to assess the psychometric properties of the questionnaire were done on the validation sample with the discharge and follow-up data pooled together, and then replicated on the full sample (construction + validation) on the discharge and follow-up data separately, to confirm that the structure held for both IBHQ versions. As the results of the validation analyses on the various samples were very similar, only results from the total sample for the discharge and follow-up questionnaires are given here, in order to provide data on the validation of both versions of the questionnaire.

Categorical variables are presented as absolute and relative frequencies, while continuous variables are presented as mean ± standard deviation. All analyses were performed using SAS statistical software version 9.2 (SAS Institute, Cary, NC, USA).

#### Finalization of the IBHQ^©^

The structure of the questionnaire was investigated using Principal Component Analysis (PCA) with PROMAX rotation [15], a technique commonly used to define item-dimension structures of questionnaires by scrutinizing the self-organization of items. The questionnaire dimensions were created from factors with an eigenvalue greater than 1 in the PCA that could be meaningfully interpreted. The loadings of items on factors in the PCA were also used to inform item selection. Although this finalization process was supported by statistical results, decisions on the items selection and creation of scores were eventually made after careful clinical interpretation based on item content and the hypothesized conceptual model.

#### Assessment of psychometric properties of the questionnaire

Reliability coefficients of the scores were estimated using Cronbach's alpha coefficient, which assesses internal consistency reliability; values greater than 0.70 were considered acceptable [15].

Construct validity was evaluated using the multitrait-multimethod approach, based on the correlations between items and dimension scores [16], i.e. item convergent validity (strong correlation of an item with its own dimension score), and item discriminant validity (correlation of an item with its own dimension score compared to all the other dimension scores).

#### Sample size

A total of 584 infants were originally planned for inclusion to allow the PCA to be applied on a construction sample of 350 discharge questionnaires. This target sample size resulted from the recommendations regarding appropriate sample sizes for factor analysis [17]. Even though this sample size was not achieved, it was possible to perform the PCA and finalize the questionnaire using a construction sample including 354 questionnaires, which were obtained by pooling 173 discharge and 181 follow-up questionnaires.

### Ethics

The study was conducted in accordance with the principles established in the Declaration of Helsinki and in compliance with local regulatory requirements. The study protocol was submitted to and approved by the board of the French National Medical Council (*Conseil national de l’ordre des médecins*) before study commencement. Study data were anonymized before data entry according to a procedure validated by the French Data Protection Authority (*Commission nationale informatique et libertés*). Only parents that returned the signed informed consent took part in the study.

## Results

### Description of the population

**Figure 1:**
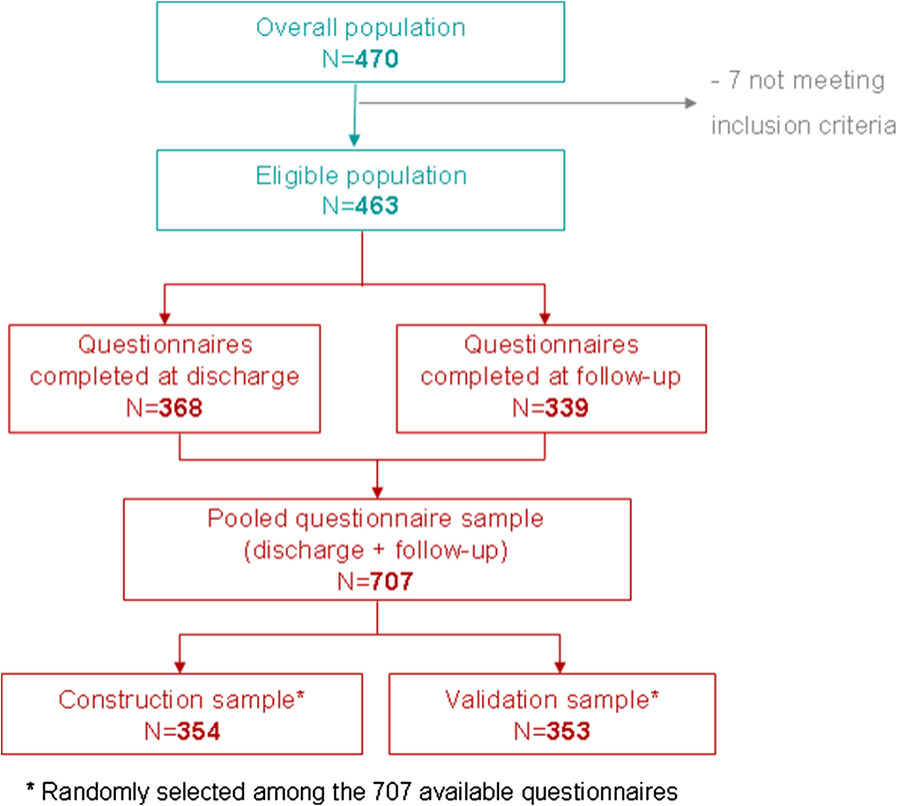
Description of analysis populations.

Four hundred and seventy infants hospitalized for bronchiolitis were included in the study. Among them, 7 did not meet one of the inclusion criteria and were not included in the analyses (Figure 1). Parents completed 707 questionnaires (368 discharge and 339 follow-up questionnaires) which were randomly divided into a construction sample (N=354) and a validation sample (N=353).

Seventy-five percent of the infants were below 5 months old, and 72.3% had at least one sibling (Table 1). The study included 332 (71.7%) full-terms, and 131 (28.3%) preterm infants; 28 (6.0%) infants had CHD. This segmentation of the study population was due to recruitment difficulties that led to the release of the initial inclusion constraint.

**Table 1:** Characteristics of the eligible infants included in the study (N=463)

**Age at hospitalization - months**	
Mean (SD)	3.3 (2.7)
Median (Q1 – Q3)	2.5 (1.3 – 5.0)
Min – Max	0.3 – 12.0
**Sex – Male n (%)**	254 (54.9)
**Siblings – n (%)**	
0	120 (25.9)
1	203 (43.8)
2	89 (19.2)
3 or more	43 (9.3)
**Child from multiple pregnancy - n (%)**	36 (7.8)
**Premature - n (%)**	131 (28.3)
**Congenital heart disease - n (%)**	28 (6.0)
**APGAR score (N=420)**	
Mean (SD)	9.2 (1.8)
Median (Q1 – Q3)	10.0 (9.0 – 10.0)
Min – Max	0.0 – 10.0

Most of the parents who completed the questionnaire were mothers; only 26 fathers (7.1%) completed the discharge questionnaire and 22 (6.7%) the follow-up questionnaire. Fathers were slightly older than mothers, and were more likely to have a full-time job (Table 2). Almost half (42.5%) of the mothers who completed the questionnaire were housewives.

**Table 2:** Characteristics of the parents who completed the questionnaire (at hospital discharge)

	**Parent who completed the questionnaire**	
	**Mother (N=308)**	**Father (N=26)**
**Age (years) – Mean (SD)**	30.1 (4.8)	33.6 (6.9)
**Level of education – n (%)**		
≤ 9 years	16 (5.2)	1 (3.9)
10–12 years	56 (18.2)	7 (26.9)
13–15 years	153 (49.7)	9 (34.6)
≥ 16 years	48 (15.6)	4 (15.4)
**Professional status – n (%)**		
Full-time employed	102 (33.1)	17 (65.4)
Part-time employed	41 (13.3)	5 (19.2)
Job seeker	17 (5.5)	1 (3.9)
Student	3 (1.0)	0 (0.0)
Disabled	0 (0.0)	1 (3.9)
Housewife	131 (42.5)	0 (0.0)

### Finalization of the IBHQ^©^

Three hundred sixty-eight (79.5%) parents returned the discharge questionnaire, and 339 (73.2%) returned the follow-up questionnaire. All items had less than 5% missing data (mean rate of 2.1% per questionnaire), except for the four items on siblings’ reactions (5-7% missing data) and the two items on breastfeeding (10-15% missing data); 580 (82.0%) questionnaires had no missing data for core items.

Six items were deleted because they did not fit the structure emerging from the PCA of the core items performed on the validation sample. The structure obtained from the remaining 30 core items included 7 factors with eigenvalues higher than 1, explaining 65% of the total variance (factor loadings of this PCA provided as Additional file 2: Table S2). These results, in light of the item content and hypothesized conceptual model, clearly supported a 7-dimension structure for the core items: “Worries and distress”; “Fear for future”; “Guilt”; “Impact on daily organization”; “Physical impact”; “Impact on behavior with hospitalized infant”; and “Financial impact”.

Structure of the optional items was informed by additional PCAs that included optional items in addition to the 30 retained core items; the structure of the core items remained stable throughout these additional PCAs. These analyses revealed that items about siblings and the infant’s reaction could each support two scores: “Impact on behavior with other children” and “Siblings’ reaction” for the former and “Physical reaction of the hospitalized infant” and “Impact on feeding of the hospitalized infant” for the latter. For breastfeeding items, the factor “Disturbed breastfeeding” was generated based on the 75 questionnaires corresponding to breastfed infants. No factor regarding the impact on the couple’s relationship was observed, and therefore the corresponding items were deleted from the questionnaire.

### IBHQ scoring rules

The final questionnaire, named Impact of Bronchiolitis Hospitalization Questionnaire (IBHQ^©^), is described in Table 3 and includes 30 core items, allowing the calculation of 7 dimension scores, as well as 16 optional items, allowing the calculation of 5 dimension scores [18]. The two separate versions of the questionnaire, for the assessment of the impact at discharge and during follow-up, were called the IBHQ-DC and IBHQ-FU, respectively.

**Table 3:** Item content, dimension structure of the IBHQ and internal consistency reliability of IBHQ-DC and IBHQ-FU – N=368 (IBHQ-DC); N=339 (IBHQ-FU)

**Type**	**Factor/Dimension**	**Item #**	**Item content**	**Cronbach’s alpha**	
				**IBHQ-DC**	**IBHQ-FU**
**Core**	Worries and distress	7	Worry about infant’s pain	0.86	0.91
			Stress		
			Worry about uncertainty		
			Helplessness		
			Demoralization		
			Panic		
			Anger		
	Fear for future	3	Fear about consequences	0.78	0.82
			Fear for infant’s life		
			Fear of other bronchiolitis		
	Guilt	3	Guilt about leaving the infant in hospital	0.61	0.64
			Guilt about bronchiolitis		
			Loneliness		
	Impact on daily organization	6	Trouble with organization of household	0.78	0.82
			Trouble with organization with family life		
			Trouble with meal habits		
			Trouble with organization of leisure activities		
			Trouble with sleeping arrangements		
			Trouble with travel to hospital		
	Physical impact	4	Sickness	0.70	0.81
			Physical tiredness		
			Sleeping disorders		
			Disturbed appetite		
	Impact on behavior with hospitalized infant	3	Increased protection	0.70	0.76
			Decreased severity		
			Increased carefulness		
	Financial impact	4	Decreased income	0.62	0.62
			Trouble with work organization		
			Increased expenses		
			Financial concern		
**Optional**	Disturbed breastfeeding	2	Disturbed breastfeeding	0.84	0.89
			Disturbed onset of lactation		
	Physical reaction of hospitalized infant	4	Crying	0.86	0.90
			Agitation		
			Grumpiness		
			Tension		
	Impact on feeding of hospitalized infant	4	Disturbed feeding	0.78	0.75
			Loss of appetite		
			Weight loss		
			Unreactive infant		
	Impact on behavior with other children	3	Hiding worry	0.65	0.78
			Worry about childcare for other child		
			Feeling of taking less care		
	Siblings’ reaction	3	Sadness because of the infant’s absence	0.79	0.83
			Worry about infant		
			Sadness because of the parents’ absence		

All scores can be obtained by summing the responses to the items related to the corresponding dimension and then applying a linear transformation resulting in a score ranging from 0 to 100, with the following formula:

$$\text{Final}\;\text{score}=\frac{\text{Raw}\;\text{score}-\text{Lowest}\;\text{possible}\;\text{score}}{\text{Raw}\;\text{score}\;\text{range}}\times 100$$

Scores were calculated only if the majority of items included in the dimension were available. All scores were designed so that 0, the lowest possible value, designates the case of parents not at all impacted, and 100, the highest possible value, corresponds to the case of parents reporting the highest impact category for all items of the dimension.

### Assessment of the psychometric properties of the IBHQ

The internal consistency reliability of the IBHQ was acceptable (Table 3): Cronbach’s alpha coefficients were above the threshold value of 0.7 for all dimensions, except “Guilt” and “Financial impact”, for which it was below 0.7 for both IBHQ-DC and IBHQ-FU, and “Impact on behavior with other infants” for which it was low for IBHQ-DC only.

Item convergent and discriminant validity was satisfactory for the great majority of scores, providing evidence supporting the construct validity of the questionnaire (detailed results provided as Additional file 3: Table S3). Few dimensions had both imperfect item convergent and discriminant validity: “Guilt” and “Financial impact” at both hospital discharge and at follow-up and “Impact on daily organization” at follow-up only. In addition, one item of “Physical impact” and “Impact on behavior with hospitalized infant” did not meet the convergent criterion at hospital discharge.

The correlations between IBHQ domain scores were moderate to low (inter-dimension correlation coefficients provided as Additional file 4: Table S4 for IBHQ-DC and as Additional file 5: Table S5 for IBHQ-FU); the only correlation coefficients greater than 0.5 both for IBHQ-DC and IBHQ-FU were observed between the three dimensions assessing psychological impact: “Fear for future”, “Worries and distress” and “Guilt”.

### Description of IBHQ dimension scores

**Figure 2:**
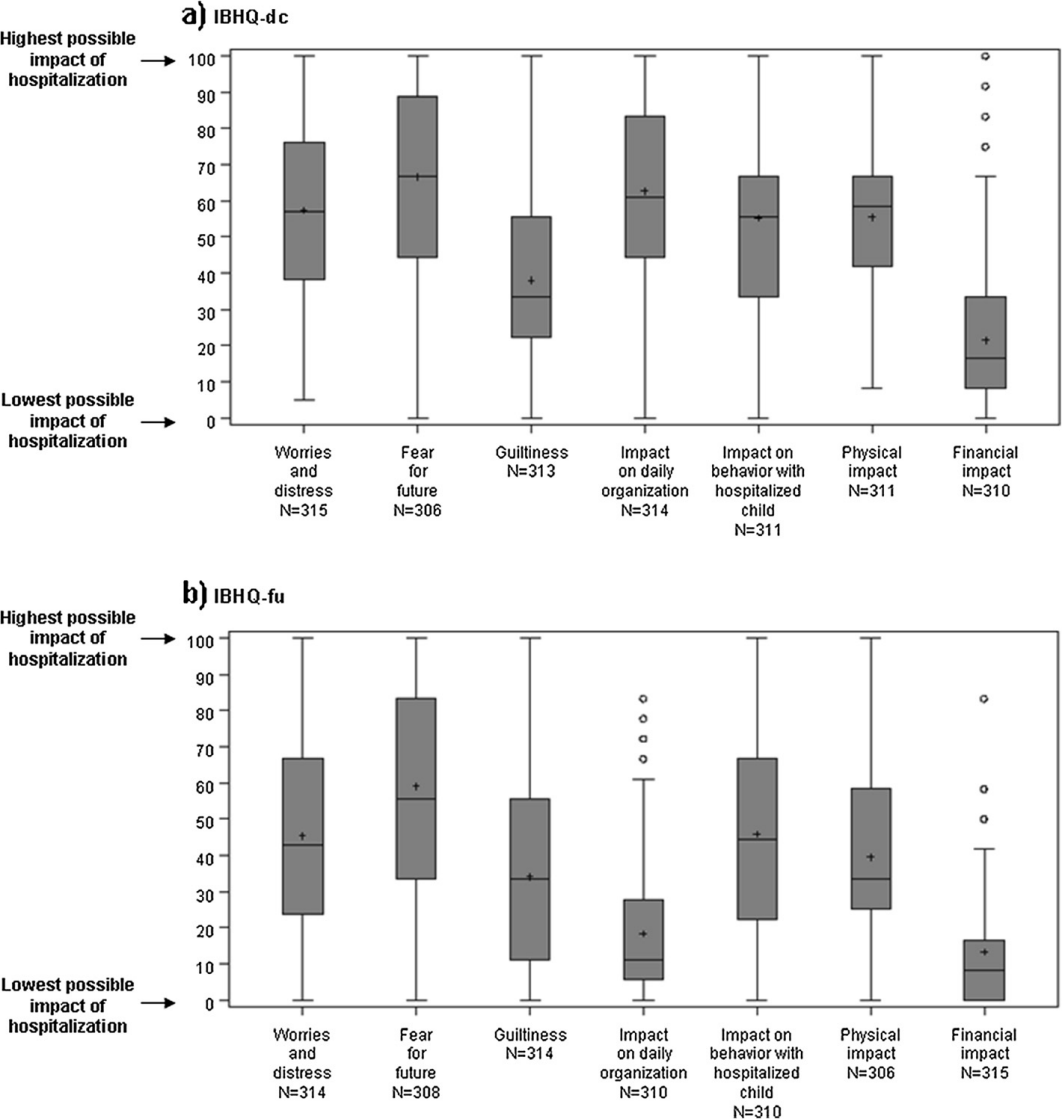
**Description of IBHQ core scores for a) IBHQ-DC and b) IBHQ-FU – Box-plots.** Box for each score: interquartile range (Q1-Q3); +: mean; –: median; bottom and top bars: observed minimum and maximum observed values; ○: outliers (i.e. values that are outside the distance of 1.5 times the interquartile range from Q1 or Q3).

**Figure 3:**
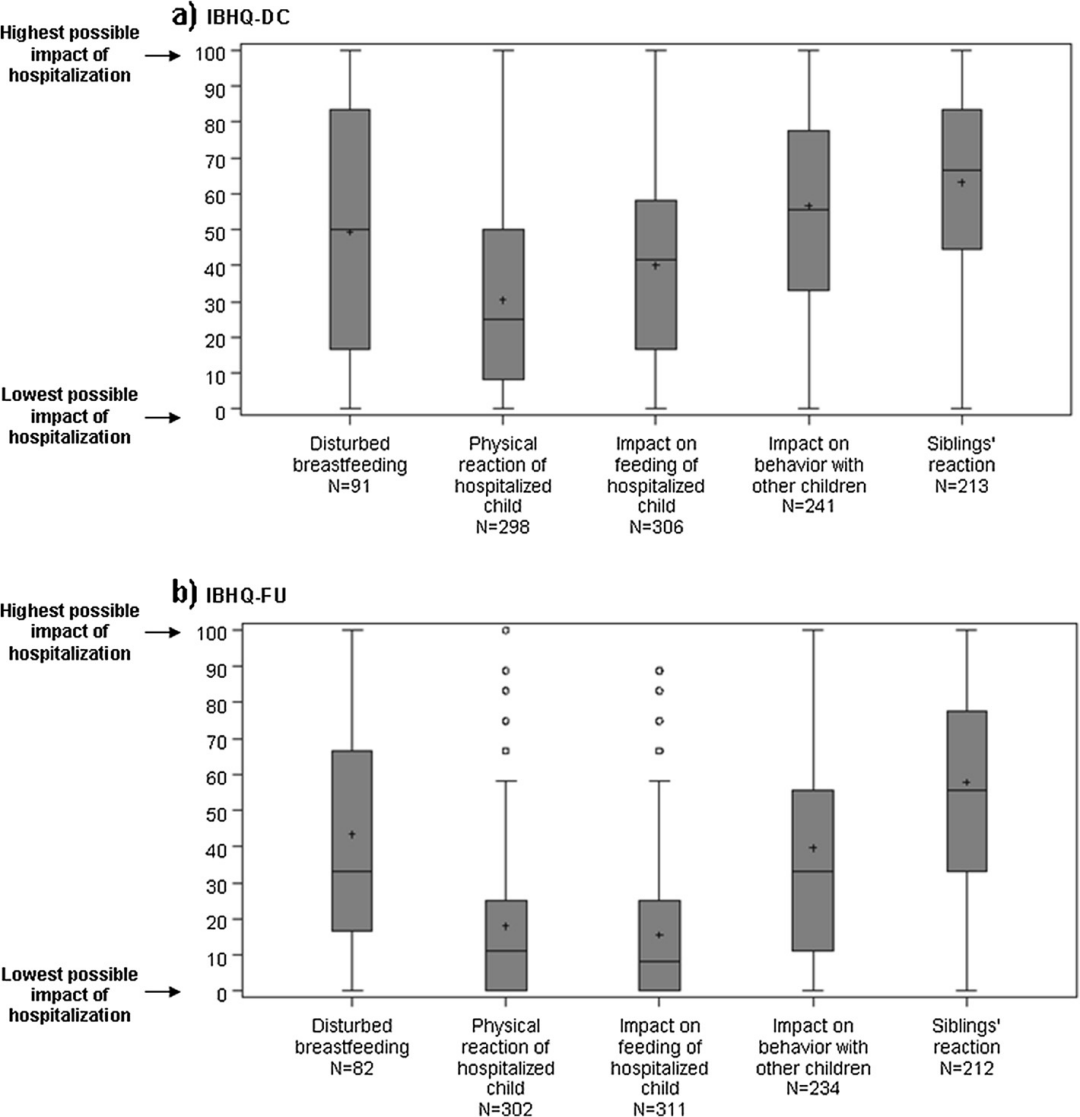
**Description of IBHQ optional scores for a) IBHQ-DC and b) IBHQ-FU – Box-plots.** Box for each score: interquartile range (Q1-Q3); +: mean; –: median; bottom and top bars: observed minimum and maximum observed values; ○: outliers (i.e. values that are outside the distance of 1.5 times the interquartile range from Q1 or Q3).

The distribution of IBHQ scores is described in Figure 2 for the core scores and in Figure 3 for the optional scores. The highest core mean scores were observed for the “Fear for future” score, both at discharge and follow-up (respective mean score±SD: 67±27 and 59±28), while the lowest were observed for the “Financial impact” score (respective mean score±SD: 21±19 and 13±14). Mean IBHQ scores were lower after 3 months than immediately after discharge.

For the optional dimensions, the highest mean scores were observed for the “Siblings’ reaction” (mean score±SD: 63±25 at hospital discharge and 58±25 at follow-up), while the lowest were observed for the “Physical reaction of hospitalized infant” (respective mean score±SD: 31±26 and 18±22) and the “Impact on feeding of hospitalized infant” (respective mean score±SD: 40±26 and 16±19). Similarly to core scores, optional scores were lower for IBHQ-FU than for IBHQ-DC.

## Discussion

The IBHQ^©^ was designed to allow the standardized assessment of the impact on parents of the hospitalization of their infant for bronchiolitis. It was developed in accordance with up-to-date reference methods [19], and it was shown to be well accepted by parents and clinicians. Even if some minor measurement issues were detected in our study, the validity and reliability of the IBHQ were overall satisfactory in a population of parents of infants hospitalized for bronchiolitis, including a significant number of infants with features specific to those that are generally hospitalized for bronchiolitis within their first year of life (i.e. preterm infants; infants issued from multiple pregnancies; infants with CHD).

The development and validation of the IBHQ highlights the multifaceted nature of the impact of hospitalization for bronchiolitis. Previous studies have shown that the impact of an infant’s illness on parents can be cognitive, psychological, social, physical and financial; hospitalization can also affect marital relationships, parenting roles, daily life and work activities [5-14]. The infant’s illness could also impact siblings and extended family. The psychometric analyses performed in our study strengthened this conclusion: the PCA clearly supported the multidimensionality of the IBHQ structure, and the low inter-score correlations indicated that IBHQ domain scores were exploring distinct aspects. This study emphasizes that the impact of the bronchiolitis hospitalization cannot be restricted to a single facet and should be considered in a comprehensive approach that encompasses a variety of factors. Moreover, this multidimensional measurement model preserved its validity 3 months after the hospitalization.

Our study showed that the great majority of the investigated aspects were impacted. The financial impact score was the only one distributed toward the lower end of the 0–100 range; all the others were distributed around the medium values of the scale or above. In particular, emotional, physical, and daily organization aspects were clearly impacted during the hospitalization. In addition, the impact on various aspects (emotional, parenting roles, and reactions of siblings) remained 3 months after hospital discharge, even if overall, the scores at follow-up were consistently lower than at discharge. These results confirmed the relevance of a questionnaire measuring all the aspects covered by the IBHQ and enabling the measurement not only at hospital discharge but also a few months later.

A previous study showed that the strong parental emotional distress associated with the infant’s hospitalization for bronchiolitis persists several weeks after discharge [20]. This previous study showed that parents and caregivers of children hospitalized for severe RSV infection exhibited remarkably high levels of anxiety during their child’s hospitalization compared to a control group (i.e. children not hospitalized for severe RSV infection and their parents/caregivers) and that these levels of anxiety were still significantly higher two months later. Of note, this previous study focused mainly on the psychological distress, and did not cover all aspects of the impact of the bronchiolitis hospitalization on parents’ life. In addition, it did not use a questionnaire to assess the impact of bronchiolitis hospitalization in a standardized manner. The results of our study are highly consistent with these data, showing the importance of scores that concern the emotional aspects (i.e. distress generated by the infant’s pain and uncertainty of his or her future, helplessness, etc.), both during hospitalization and three months after hospital discharge. But our results also enable a more complete picture of the impact on parents of the bronchiolitis hospitalization to be drawn. Further in-depth analyses of the impact on the parents of infants hospitalized for bronchiolitis as measured by the IBHQ in our study sample are reported elsewhere [21].

The demonstration of the IBHQ psychometric properties in the present study has some limitations. First, even if the IBHQ fits perfectly with the picture of a multifaceted impact on parents of the hospitalization for bronchiolitis, which was consistently shown by all research on this question, and its content validity is underpinned by the involvement of clinicians and parents throughout its development, the amount of evidence on its construct validity is still limited and should be confirmed in future research. Second, given the small number of fathers having completed the questionnaires, no reliable analysis could be performed to evaluate neither the validity of the IBHQ in fathers nor the potential differences in the impact of hospitalization depending on parent’s gender. Third, parents were required to be able to complete the questionnaire in French without help to be included in the study. This may have led to underrepresentation of parents with a lower level of education, despite this population being of particular interest since low parental education level is a documented risk factor of hospitalization for bronchiolitis [22]. The validity of the IBHQ in this specific population should be further explored. However, the validity of the self-administered version of the IBHQ may be challenging to ascertain in this population, which might benefit from having the questionnaire administered by an interviewer. Finally, the IBHQ has been developed and validated in France; as for any questionnaire, its validity in other countries with different cultures, health care, and health insurance systems, remains to be studied. To allow such studies to be conducted, a linguistic validation has been performed to make the IBHQ available in US English.

Yet the IBHQ is already a promising tool that could be used in several contexts: (i) in epidemiological studies, to explore the impact of bronchiolitis hospitalization in different populations; (ii) to identify the risk factors of strong impact, thus helping clinicians to focus on those parents who could benefit from maximal support during this stressful experience; (iii) to evaluate innovative health care strategies for bronchiolitis in light of their benefit for the parents.

## Conclusions

The IBHQ is a unique instrument assessing the impact on parents of the hospitalization of their infant for bronchiolitis. It was developed according to state-of-the-art methods and demonstrated good measurement properties in a study with a significant number of subjects.

The impact on parents of their infant’s hospitalization for bronchiolitis was thus demonstrated to be multifaceted. The organizational, emotional and physical consequences of the hospitalization of an infant for bronchiolitis may persist several months after discharge, and should not be underestimated.

## Competing interests

AL, VG, JBG and GM received an honorarium from AbbVie for their scientific advice and expertise. AR, KB and BA are employees of Mapi, consulting company commissioned by AbbVie for this study. DA is an employee of AbbVie, and may own AbbVie Stock. AbbVie participated in the design and conduct of the study, interpretation of data, review, and approval of the manuscript.

## Authors’ contributions

AL, VG, JBG and GM provided clinical and scientific expertise on the bronchiolitis disease along the project, in particular for definition of objectives, patient inclusion and non-inclusion criteria validation, patient recruitment, interpretation of results, choice of concepts to be measured, and finalization of the questionnaire, and critically reviewed the manuscript. AR participated in the study design, designed statistical analyses, participated in the interpretation of results, and drafted the manuscript. KB managed the global organization of the project, led the item generation of the questionnaire, and participated in the development of the manuscript. DA participated in the interpretation of data; critically reviewed the manuscript. BA provided scientific expertise on the methodology used to develop the questionnaire and on the design of the study and of the statistical analysis, participated in the item generation of the questionnaire and in the interpretation of the study results, and critically reviewed the manuscript. All authors read and approved the final manuscript.

## Pre-publication history

The pre-publication history for this paper can be accessed here:

http://www.biomedcentral.com/1472-6963/13/272/prepub

## Supplementary Material

Additional file 1: Table S1 Characteristics of the parents interviewed during the development of the Impact of Bronchiolitis Hospitalization Questionnnaire. Click here for file

Additional file 2: Table S2 Rotated factor pattern – PCA on the 30 core items of the Impact of Bronchiolitis Hospitalization Questionnaire retained in the final structure (Construction sample; N=289). Click here for file

Additional file 3: Table S3 Item convergent and discriminant validity and internal consistency reliability of the IBHQ-DC and IBHQ-FU. Click here for file

Additional file 4: Table S4 Inter-dimension correlation coefficients of IBHQ dimensions in the overall construction and validation sample for IBHQ-DC (N=368). Click here for file

Additional file 5: Table S5 Inter-dimension correlation coefficients of IBHQ dimensions in the overall construction and validation sample for IBHQ-FU (N=339). Click here for file

## Copyrights

The Impact of Bronchiolitis Hospitalization Questionnaire (IBHQ^©^) is protected by copyright with all rights reserved to AbbVie France. Do not use without permission. For information on, or permission to use IBHQ^©^, please contact the Mapi Research Trust, 27 rue de la Villette 69003 Lyon, France.

Tel: +33 (0) 472 13 65 75 – E-mail: trust@mapi.fr – website: http://www.mapi-trust.org.
