# Supplementary material for: Development of a questionnaire to assess the impact on parents of their infant’s bronchiolitis hospitalization
Source: BMC Health Serv Res. 2013 Jul 12;13:272. doi: 10.1186/1472-6963-13-272 (PMC3717097; doi:10.1186/1472-6963-13-272)
Supplement: Additional file 1: Table S1 — Characteristics of the parents interviewed during the development of the Impact of Bronchiolitis Hospitalization Questionnnaire. [file 1472-6963-13-272-S1.doc]

E-Table 1 Characteristics of the parents interviewed during the development of the Impact of Bronchiolitis Hospitalization Questionnnaire

| **Characteristic** | | **Stage 1 parent exploratory interviews (N=5)** | **Stage 2 parent exploratory interviews (N=16)** | **Comprehension tests (N=9)** |
| --- | --- | --- | --- | --- |
| **Parent(s) interviewed** | Father (n) | 2 | 0 | 0 |
|  | Mother (n) | 3 | 10 | 7 |
|  | Couple (n) | 0 | 3 | 2 |
| **Age** | Mean (years) | 37.2 | 32.2 | NC |
|  | Min – max (years) | 32 - 43 | 27 - 42 | NC |
| **Education level** | Secondary school (n) | 1 | 4 | NC |
|  | A-level (n) | 2 | 0 | NC |
|  | University (n) | 2 | 12 | NC |
| **Work status** | Full time (n) | 2 | 6 | NC |
|  | Part time (n) | 0 | 3 | NC |
|  | Maternity leave / Parental leave (n) | 1 | 4 | NC |
|  | Housewife (n) | 2 | 2 | NC |
|  | Unemployed (n) | 0 | 1 | NC |
| **Environment** | Urban (n) | NC | 15 | 3 |
|  | Rural (n) | NC | 1 | 2 |
|  | Missing data (n) | NC | 0 | 4 |
| **Children at home** | Mean | 2.8 | 2.3 | 3.1 |
| **Gestational age** | Full term (≥ 37 weeks) (n) | 3 | 4 | 3 |
|  | Premature (32 - 36 weeks) (n) | 0 | 5 | 3 |
|  | Highly premature (< 32 weeks) (n) | 2 | 5 | 3 |
| **Hospitalization duration** | Days | 2 to 18 | 3 to 15 | NC |

NC: not collected
