# Supplementary material for: Development of a questionnaire to assess the impact on parents of their infant’s bronchiolitis hospitalization
Source: BMC Health Serv Res. 2013 Jul 12;13:272. doi: 10.1186/1472-6963-13-272 (PMC3717097; doi:10.1186/1472-6963-13-272)
Supplement: Additional file 2: Table S2 — Rotated factor pattern – PCA on the 30 core items of the Impact of Bronchiolitis Hospitalization Questionnaire retained in the final structure (Construction sample; N=289). [file 1472-6963-13-272-S2.doc]

E-Table 2 Rotated factor pattern – PCA on the 30 core items of the Impact of Bronchiolitis Hospitalization Questionnaire retained in the final structure (Construction sample; N=289)

| **Hypothesized dimension - Item** | **Factor 1** | **Factor 2** | **Factor 3** | **Factor 4** | **Factor 5** | **Factor 6** | **Factor 7** |
| --- | --- | --- | --- | --- | --- | --- | --- |
| Emotional - Worry about infant’s pain | 0.85 | -0.01 | -0.01 | -0.20 | 0.10 | -0.06 | 0.13 |
| Emotional - Stress | 0.83 | -0.01 | 0.10 | -0.05 | 0.07 | -0.02 | -0.09 |
| Emotional - Worry about uncertainty | 0.76 | 0.02 | -0.14 | -0.07 | 0.06 | 0.04 | -0.01 |
| Emotional – Helplessness | 0.74 | 0.17 | -0.02 | 0.00 | -0.07 | 0.03 | 0.00 |
| Emotional – Demoralization | 0.73 | -0.02 | 0.19 | 0.13 | -0.07 | 0.18 | -0.08 |
| Emotional – Panic | 0.69 | -0.14 | 0.19 | 0.16 | 0.00 | 0.15 | -0.07 |
| Emotional - Fear about consequences | 0.56 | 0.12 | -0.18 | 0.12 | -0.08 | -0.05 | 0.42 |
| Emotional – Anger | 0.54 | -0.18 | 0.21 | 0.19 | -0.05 | 0.17 | -0.04 |
| Emotional - Fear for infant’s life | 0.51 | -0.02 | -0.01 | 0.00 | 0.10 | -0.17 | 0.48 |
| Emotional - Fear of other bronchiolitis | 0.48 | -0.05 | -0.03 | 0.18 | -0.02 | -0.16 | 0.37 |
| Daily organization - Trouble with organization of household | 0.03 | 0.92 | -0.08 | 0.00 | 0.01 | 0.04 | -0.01 |
| Daily organization - Trouble with organization of family life | 0.05 | 0.89 | -0.01 | 0.05 | -0.05 | 0.05 | -0.04 |
| Daily organization - Trouble with meal habits | 0.08 | 0.80 | -0.03 | 0.01 | -0.01 | 0.11 | -0.02 |
| Daily organization - Trouble with organization of leisure activities | -0.01 | 0.76 | 0.12 | -0.08 | 0.01 | 0.04 | 0.07 |
| Daily organization - Trouble with sleeping arrangements | -0.10 | 0.76 | 0.15 | 0.02 | 0.13 | -0.08 | -0.12 |
| Daily organization - Trouble with travel to hospital | -0.15 | 0.66 | 0.05 | 0.14 | -0.09 | 0.14 | 0.28 |
| Physical - Sickness | -0.01 | -0.11 | 0.75 | -0.11 | -0.01 | -0.02 | 0.19 |
| Physical – Physical tiredness | -0.06 | 0.27 | 0.72 | 0.07 | 0.06 | -0.22 | 0.02 |
| Physical - Sleeping disorders | 0.15 | 0.25 | 0.70 | -0.07 | 0.01 | -0.21 | 0.03 |
| Physical - Disturbed appetite | 0.33 | 0.24 | 0.48 | 0.03 | -0.10 | 0.00 | 0.10 |
| Emotional - Guilt about leaving in hospital | 0.03 | 0.01 | -0.08 | 0.78 | 0.00 | -0.05 | 0.02 |
| Emotional - Guilt about bronchiolitis | 0.11 | 0.09 | -0.14 | 0.73 | 0.03 | -0.15 | 0.02 |
| Emotional - Loneliness | -0.06 | 0.01 | 0.18 | 0.65 | 0.01 | 0.17 | -0.01 |
| Behavior with children – Increased protection | 0.13 | -0.01 | 0.01 | 0.12 | 0.77 | -0.03 | 0.01 |
| Behavior with children – Decreased severity | -0.02 | 0.01 | -0.01 | -0.21 | 0.75 | 0.09 | 0.08 |
| Behavior with children – Increased Carefulness | 0.01 | 0.02 | 0.01 | 0.28 | 0.74 | 0.04 | -0.02 |
| Financial – Decreased income | 0.02 | 0.02 | -0.12 | 0.02 | 0.02 | 0.86 | 0.24 |
| Daily organization - Trouble with organization of work | 0.12 | 0.36 | -0.17 | -0.10 | 0.08 | 0.70 | -0.06 |
| Financial - Increased expenses | -0.05 | 0.12 | 0.26 | -0.13 | 0.07 | 0.25 | 0.64 |
| Financial – Financial concern | -0.10 | -0.15 | 0.36 | 0.10 | 0.01 | 0.33 | 0.60 |
