# Supplementary material for: Development of a questionnaire to assess the impact on parents of their infant’s bronchiolitis hospitalization
Source: BMC Health Serv Res. 2013 Jul 12;13:272. doi: 10.1186/1472-6963-13-272 (PMC3717097; doi:10.1186/1472-6963-13-272)
Supplement: Additional file 3: Table S3 — Item convergent and discriminant validity and internal consistency reliability of the IBHQ-DC and IBHQ-FU. [file 1472-6963-13-272-S3.doc]

E-Table 3 Item convergent and discriminant validity and internal consistency reliability of the IBHQ-DC and IBHQ-FU

|  |  | **Dimension score^b^** | **Range of item-scale correlations** | **Convergent validity criterion^c^ (% of items)** | **Discriminant validity criterion^d^ (% of items)** |
| --- | --- | --- | --- | --- | --- |
| IBHQ-DC  (N=368) | Core items (N=346)^a^ | WD | 0.46-0.76 | 100% | 100% |
|  |  | FF | 0.59-0.69 | 100% | 100% |
|  |  | GU | 0.32-0.52 | 67% | 100% |
|  |  | DO | 0.43-0.67 | 100% | 100% |
|  |  | PI | 0.33-0.55 | 75% | 100% |
|  |  | BC | 0.36-0.63 | 67% | 100% |
|  |  | FI | 0.36-0.55 | 50% | 100% |
|  | Infant’s reaction (N=357)^a^ | PR | 0.63-0.74 | 100% | 100% |
|  |  | IF | 0.40-0.76 | 100% | 100% |
|  | Siblings (N=248)^a^ | IB | 0.47-0.58 | 100% | 100% |
|  |  | SR | 0.58-0.76 | 100% | 100% |
| IBHQ- FU  (N=339) | Core items (N=315)^a^ | WD | 0.59-0.80 | 100% | 100% |
|  |  | FF | 0.63-0.72 | 100% | 100% |
|  |  | GU | 0.40-0.52 | 67% | 67% |
|  |  | DO | 0.39-0.76 | 83% | 83% |
|  |  | PI | 0.45-0.73 | 100% | 100% |
|  |  | BC | 0.42-0.68 | 100% | 100% |
|  |  | FI | 0.25-0.50 | 75% | 75% |
|  | Infant’s reaction (N=330)^a^ | PR | 0.76-0.80 | 100% | 100% |
|  |  | IF | 0.41-0.78 | 100% | 100% |
|  | Siblings (N=227)^a^ | IB | 0.55-0.67 | 100% | 100% |
|  |  | SR | 0.62-0.76 | 100% | 100% |

^a^ Number of questionnaires with at least 50% of items completed

^b^ WD, Worries and Distress; FF, Fear for Future; GU, Guilt; DO, impact on Daily Organization; PI, Physical Impact; BC, impact on Behavior with hospitalized Infant; FI, Financial Impact, BF, Breast Feeding; PR, Physical Reaction of hospitalized infant; IF, Impact on Feeding of hospitalized infant; IB, Impact on Behavior with other children; SR, Siblings' Reaction

^c^ Convergent validity criterion met if item-scale correlation greater than 0.4;

^d^ Discriminant validity criterion met if the correlation of an item with its own scale is greater than the correlation of this item with all the other scales
