# Supplementary material for: Development of a questionnaire to assess the impact on parents of their infant’s bronchiolitis hospitalization
Source: BMC Health Serv Res. 2013 Jul 12;13:272. doi: 10.1186/1472-6963-13-272 (PMC3717097; doi:10.1186/1472-6963-13-272)
Supplement: Additional file 5: Table S5 — Inter-dimension correlation coefficients of IBHQ dimensions in the overall construction and validation sample for IBHQ-FU (N=339). [file 1472-6963-13-272-S5.doc]

E-Table 5 Inter-dimension correlation coefficients of IBHQ dimensions in the overall construction and validation sample for IBHQ-FU (N=339)

| **IBHQ dimension^a^** | | **WD** | **FF** | **GU** | **DO** | **PI** | **BC** | **FI** | **BF** | **PR** | **IF** | **IB** | **SR** |
| --- | --- | --- | --- | --- | --- | --- | --- | --- | --- | --- | --- | --- | --- |
| **Core** | **WD** | 1.00 | 0.62 | 0.58 | 0.36 | 0.53 | 0.45 | 0.29 | 0.11 | 0.26 | 0.27 | 0.35 | 0.43 |
|  | **FF** | - | 1.00 | 0.49 | 0.43 | 0.51 | 0.45 | 0.34 | 0.09 | 0.23 | 0.33 | 0.27 | 0.42 |
|  | **GU** | - | - | 1.00 | 0.24 | 0.42 | 0.34 | 0.19 | -0.05 | 0.25 | 0.14 | 0.33 | 0.28 |
|  | **DO** | - | - | - | 1.00 | 0.48 | 0.44 | 0.49 | 0.19 | 0.35 | 0.42 | 0.38 | 0.24 |
|  | **PI** | - | - | - | - | 1.00 | 0.40 | 0.43 | 0.04 | 0.23 | 0.44 | 0.41 | 0.36 |
|  | **BC** | - | - | - | - | - | 1.00 | 0.30 | 0.22 | 0.44 | 0.25 | 0.32 | 0.26 |
|  | **FI** | - | - | - | - | - | - | 1.00 | 0.21 | 0.26 | 0.28 | 0.30 | 0.09 |
| **Breastfeeding** | **BF** | - | - | - | - | - | - | - | 1.00 | 0.34 | 0.22 | 0.10 | 0.21 |
| **Infant’s reaction** | **PR** | - | - | - | - | - | - | - | - | 1.00 | 0.40 | 0.17 | 0.17 |
|  | **IF** | - | - | - | - | - | - | - | - |  | 1.00 | 0.16 | 0.25 |
| **Siblings** | **IB** | - | - | - | - | - | - | - | - | - | - | 1.00 | 0.42 |
|  | **SR** | - | - | - | - | - | - | - | - | - | - | - | 1.00 |

^a^ WD, Worries and Distress; FF, Fear for Future; GU, Guilt; DO, impact on Daily Organization; PI, Physical Impact; BC, impact on Behavior with hospitalized Infant; FI, Financial Impact, BF, Breast Feeding; PR, Physical Reaction of hospitalized infant; IF, Impact on Feeding of hospitalized infant; IB, Impact on Behavior with other children; SR, Siblings' Reaction
